# A novel Carcinoembryonic Antigen (CEA)-Targeted Trimeric Immunotoxin shows significantly enhanced Antitumor Activity in Human Colorectal Cancer Xenografts

**DOI:** 10.1038/s41598-019-48285-z

**Published:** 2019-08-12

**Authors:** R. Lázaro-Gorines, J. Ruiz-de-la-Herrán, R. Navarro, L. Sanz, L. Álvarez-Vallina, A. Martínez-del-Pozo, J. G. Gavilanes, J. Lacadena

**Affiliations:** 1 Departamento de Bioquímica y Biología Molecular, Facultad de Ciencias Químicas, Universidad Complutense de Madrid, Avenida Complutense s/n, Madrid, 28040 Spain; 2 Unidad de Inmunología Molecular, Hospital Universitario Puerta de Hierro, Joaquín Rodrigo 2, Majadahonda, 28222 Madrid, Spain; 3 Immunotherapy and Cell Engineering, Department of Engineering, Aarhus University, Gustav Wieds Vej 10, Aarhus, 8000C Denmark

**Keywords:** Immunotherapy, Targeted therapies, Cancer immunotherapy

## Abstract

Immunotoxins are chimeric molecules, which combine antibody specificity to recognize and bind with high-affinity tumor-associated antigens (TAA) with the potency of the enzymatic activity of a toxin, in order to induce the death of target cells. Current immunotoxins present some limitations for cancer therapy, driving the need to develop new prototypes with optimized properties. Herein we describe the production, purification and characterization of two new immunotoxins based on the gene fusion of the anti-carcinoembryonic antigen (CEA) single-chain variable fragment (scFv) antibody MFE23 to α-sarcin, a potent fungal ribotoxin. One construct corresponds to a conventional monomeric single-chain immunotoxin design (IMTXCEAαS), while the other one takes advantage of the trimerbody technology and exhibits a novel trimeric format (IMTXTRICEAαS) with enhanced properties compared with their monomeric counterparts, including size, functional affinity and biodistribution, which endow them with an improved tumor targeting capacity. Our results show the highly specific cytotoxic activity of both immunotoxins *in vitro*, which was enhanced in the trimeric format compared to the monomeric version. Moreover, the trimeric immunotoxin also exhibited superior antitumor activity *in vivo* in mice bearing human colorectal cancer xenografts. Therefore, trimeric immunotoxins represent a further step in the development of next-generation therapeutic immunotoxins.

## Introduction

Antibody-based biological drugs constitute one of the main therapeutic strategies against cancer^1,2^. Currently, there are nearly 30 monoclonal antibodies approved for use in cancer immunotherapy^3–6^. Antibody design has evolved to improve their stability, half-life, and solid tumor penetration, along with the incorporation of bioactive payloads to increase the therapeutic effect. This implies new formats such as single-chain variable fragments (scFv), single-domain antibodies (sdAb), diabodies, and other multivalent designs^5,7–9^. Immunotoxins have emerged as potent and promising antitumor agents in the cancer immunotherapy field, due to their high cytotoxicity, specificity and effectivity^3,10–17^. These chimeric molecules comprise a targeting domain, usually antibody fragments that guide the action of a protein or non-protein toxin (toxic domain) and are designed to trigger specific cell death upon recognition of a tumor-associated antigen (TAA) on the surface of the target cells^18^. Their mechanism of action involves TAA binding by the targeting domain, internalization of the complex by endocytosis, and subsequent release of the toxic domain that will cause target cell death^10,11,18,19^. Thereby, the antitumor effect of the immunotoxin depends on several factors: the specificity and functional affinity of the antibody for the cell surface expressed TAA, the complex internalization effectiveness and the speed of the toxin release pathway and its own intrinsic potency and specificity.

Whereas the first immunotoxins were constructed using chemical conjugation based on full-length mAbs, currently most of them are designed as recombinant fusion proteins based on antibody fragments, linked by a flexible peptide (scFv), a disulfide bridge (dsFv) or both (scdsFv), with reduced size and improved tumor penetration, to achieve greater specificity and antitumor efficacy^11,20–25^. In fact, the first FDA-approved protein-based immunotoxin (September 2018) was moxetumomab pasudotox, composed of a scFv anti-CD22 fused to a truncated form of *Pseudomonas* exotoxin A, indicated for the treatment of hairy cell leukemia^26^.

Going further, the antibody engineering field has driven the development of a myriad of new formats with improved properties, which potentially can be included within immunotoxin constructs^27^. This new generation of antibody formats overcomes some of the scFv limitations, mainly lower tumor retention due to its monovalence, and fast blood clearance given its size under the glomerular filtration threshold (70 kDa)^28^. Those antibodies, optimized for diagnosis and tumor therapy, should fill into a pharmacological window where medium-sized (70 to 120 kDa) and multivalent molecules would be included. Within this group, trimerbody-based molecules appear clearly highlighted because of its antigen binding and pharmacological properties^29,30^.

Trimerbodies are trivalent antibodies formed by fusing a collagen XVIII-derived homotrimerization (TIE^XVIII^) domain to the C-terminal edge of a scFv or sdAb^5,27,31^. This TIE^XVIII^ domain and the antibody are directly joined by linkers of different lengths, which provide the necessary flexibility for the intended use^29,32^. Within this construct, the TIE^XVIII^ domain confers a trimeric state to the fused scFv or sdAb in solution^5,27,33^. In addition, trimerbodies have high-stability in human serum, which added to their simple design, turn them in an ideal antibody format for tumor detection and therapy^8,27,30^. In this sense, bispecific hexavalent trimerbodies have been produced and demonstrated to be completely functional^8,32^.

Regarding the toxic moiety, multiple types of toxins with different origins, have been employed such as the plant toxin ricin or the above-mentioned *Pseudomonas* exotoxin A^34–37^. Nowadays, ribonucleases from human or fungal origin have acquired a significant importance as components of the toxic domain^38–40^. In this sense, within the family of extracellular fungal ribonucleases, ribotoxins stand out for their potential use as part of immunotoxins because of their small size, high thermostability, poor immunogenicity, resistance to proteases, and their highly efficient ability to inactivate ribosomes^19,25,41–44^. As proved by its functional properties and previous uses within immunotoxins, α-sarcin seems to be one of the most promising candidate to be included in immunotoxin therapeutic designs^16,19,25,45,46^. Its specific ribonucleolytic activity against one single bond, strategically located at the large rRNA, causes ribosome inactivation and, thereby, protein biosynthesis inhibition and apoptosis^47–49^.

In this study, the targeted TAA is carcinoembryonic antigen (CEA), also known as CEACAM5, and originally described as an oncofetal protein in colorectal cancer^50^. It is present at low levels in adult tissues from epithelial origin, such as colon, stomach, tongue, cervix and prostate, being its overexpression and the change in its pattern of expression indicative of tumor transformation^51^. While in non-tumor cells CEA is restricted to the apical surface, in cancerous cells it appears dislocated all over the cellular membrane, contributing to its secretion^52^. CEA is constitutively released from tumor cells reaching detectable concentrations in peripheral blood. In fact, CEA quantification has been frequently used with diagnosis and prognosis purposes^52–54^. CEA represents a potential target for immunotoxins, due to its internalization and half-life between 10 and 16 h^53^. MFE-23 was the first anti-CEA scFv to be used in patients, precisely to target colorectal cancer in imaging and antibody-directed prodrug therapy strategies^8,27,33,55^. Thereby, these data support the use of CEA as a validated target for colorectal cancer and the MF23 scFv as the binding domain for anti-CEA immunotoxins.

Accordingly, we herein describe the production and characterization of two new immunotoxins: monomeric IMTXCEAαS and trimeric IMTXTRICEAαS. The monomeric version is based on the design of IMTXA33αS, an immunotoxin containing α-sarcin and directed against the colorectal-associated glycoprotein A33 (GPA33), with potent antitumor activity *in vivo*^16,19,25,45^. On the other hand, IMTXTRICEAαS was designed according to a trimerbody format, including the human TIE^XVIII^ trimerization domain, flanked by an 18 amino acid linker^27^. Trimerbodies based on MFE23 scFv have been produced in mammalian and *P*. *pastoris* cells^8,27,33^, showing functional binding to CEA *in vitro* and imaging of CEA-positive xenografts in *nude* mice^27,55,56^. The results shown here, not only prove the antitumoral activity of these new constructs, but also reveal the highly improved toxicity of the trimeric design over its monomeric counterpart. Together, they open up a new avenue of development in the field of specific antitumor immunotoxins.

## Results

### Generation, production and purification of the immunotoxins

**Figure 1:**
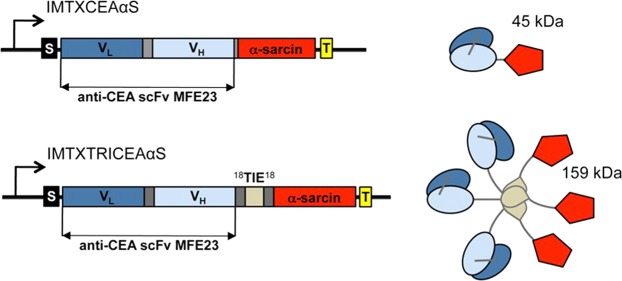
Schematic diagrams showing the genetic and domain arrangements of both immunotoxin designs: IMTXCEAαS and IMTXTRICEAαS. The cDNA constructs employed are shown at the left part of the figure. The right side shows the schematic representation of the native proteins displaying their different domains arrangement. In both cases (cDNAs and proteins), structural and functional motifs are highlighted with colours: α-factor secretion signal peptide (S, black); scFvCEA (V_L_, dark blue, and V_H_, light blue), L18 linkers (^18^, dark gray); TIE^XVIII^, human non-colagenous trimerization domain (TIE, light gray); α-sarcin (red) and histidine-tag (T, yellow). Given its trimeric structure, native IMTXTRICEAαS should display a theoretical size of 159 kDa, meanwhile for monomeric IMTXCEAαS the expected mass value should be 45 kDa.

**Figure 2:**
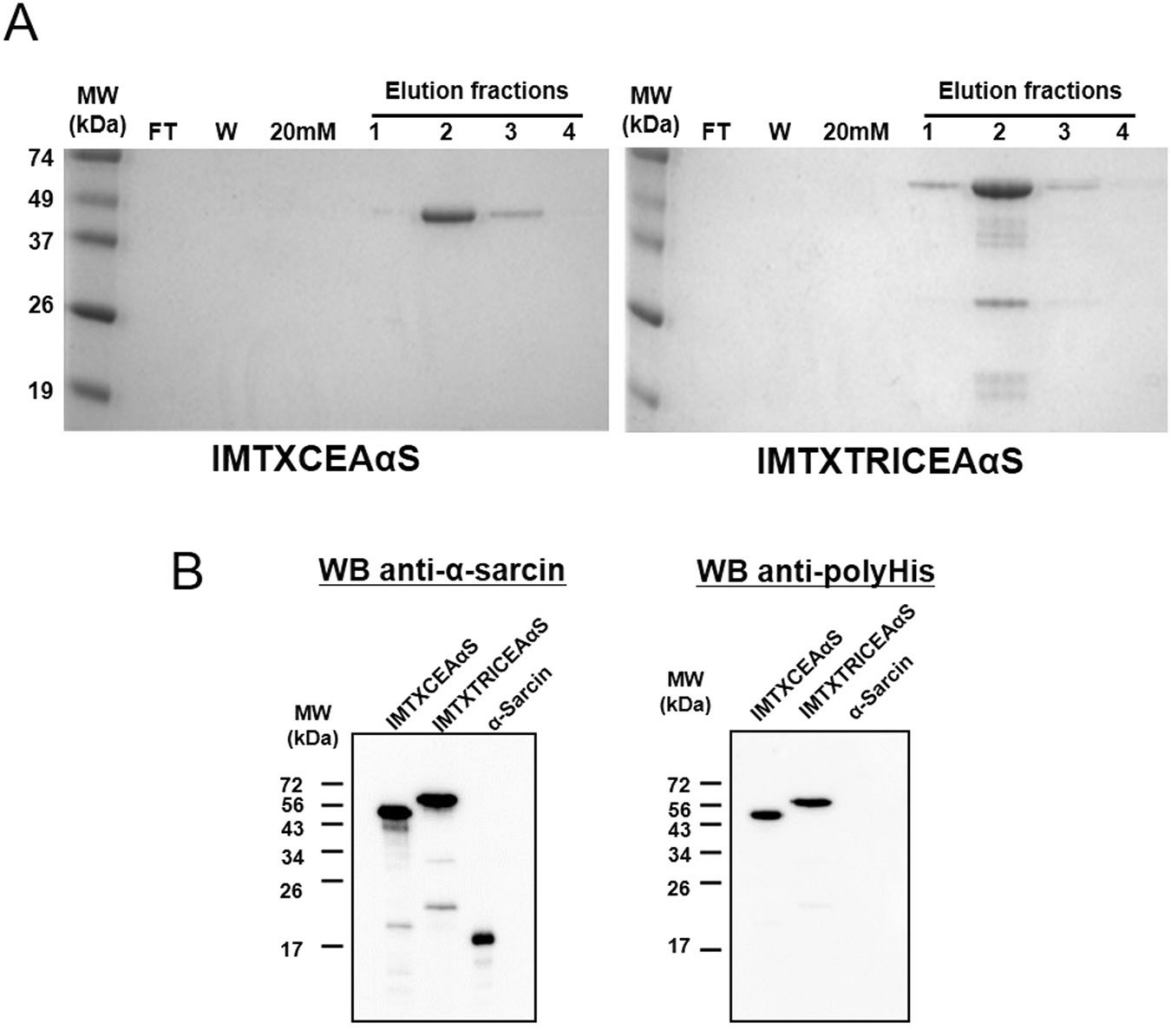
SDS-PAGE and Western Blot analysis of both purified immunotoxins. (**A**) Coomassie blue stained SDS-PAGE analysis of the different pools obtained during the Ni^2+^-NTA affinity chromatography employed for the immunotoxins purification. Lines shown correspond to: FT, not retained fraction; W, washed fraction eluted with chromatography buffer (see Methods), 20 mM, washed fraction eluted with the same chromatography buffer but containing 20 mM imidazole; and the first four 1 ml fractions eluted with 250 mM imidazole. (**B**) Western blot analysis using rabbit anti-α-sarcin serum (left) or commercial anti-histidine tag antibody (right). α-Sarcin designates 0.1 µg of the fungal natural protein used as a control. MW corresponds to prestained Bio-Rad Precision Plus protein molecular weight standards. Images correspond to full-length gels and blots acquired and analyzed using the Gel Doc XR Imaging System and Quantity One 1-D analysis software (BioRad) or ChemiDoc-It (UVP) and VisionWorks LS, respectively. Different exposures of the blots are presented in Supplementary Fig. S1.

Expression vectors for both immunotoxins were generated, as described in the Methods section (Fig. 1) and were successfully produced in *P*. *pastoris*. The immunotoxins were purified from the extracellular media, following dialysis and immobilized metal affinity chromatography. Then, the identity and homogeneity of the isolated proteins were analyzed by SDS-PAGE and western blot immunodetection (Fig. 2). Bands of the expected theoretical molecular weight, visualized by means of Coomassie Blue staining, were also specifically recognized by the anti-α-sarcin serum. The expected masses were 45 and 53 kDa for IMTXCEAαS and IMTXTRICEAαS monomers, respectively, in reducing and denaturing conditions. The purification yield of both proteins was around 1 mg/l of induction media.

### Structural characterization

**Figure 3:**
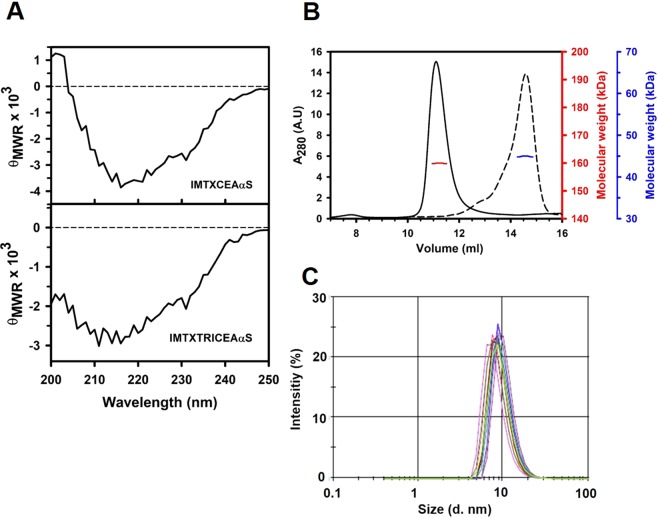
Structural characterization. (**A**) Far-UV CD spectra of IMTXCEAαS and IMTXTRICEAαS are shown (*θ*_MRW_, mean residue weight ellipticities expressed as degree × cm^2^ × dmol^−1^). Both spectra were performed in 50 mM sodium phosphate, 0.1 M NaCl pH 7.4 at a protein concentration of 0.15 mg/ml. (**B**) Analysis of the monomeric or oligomeric nature of IMTXCEAαS (dashed line) and IMTXTRICEAαS (solid line) was analyzed by Superdex 200 FPLC chromatography analysis. The eluted proteins showed a single symmetric elution peak at the expected volume for its predicted 45 kDa and (blue axis) 159 kDa (red axis) weight, respectively. (**C**) Laser scattering size determination of IMTXTRICEAαS reveals the presence of a globular structure with a hydrodynamic size of around 10 nm. Multiple spectra were recorded with samples at two different protein concentrations (0.15 or 0.3 mg/ml) in sodium phosphate buffer.

Both IMTXCEAαS and IMTXTRICEAαS far-UV circular dichroism (CD) spectra were fully compatible with water-soluble globular proteins with a high content of β-sheet secondary structure (Fig. 3A). Results are fully coherent with the nature and content of the secondary structures of their native components, α-sarcin and MFE23 scFv^33,57^. Differences observed between both spectra were easily explained by the presence of the 18-residue linkers and the TIE^XVIII^ domain connecting both targeting and toxic domains in the trimeric immunotoxin.

The molecular size in solution of native IMTXCEAαS and IMTXTRICEAαS was determined by FPLC size exclusion chromatographic analysis using a Superdex200 10/300 GL column. As can be observed in Fig. 3B, the elution profile of IMTXCEAαS showed a mass of 45 kDa which corresponds to its expected monomeric globular structure, while the elution profile for IMTXTRICEAαS showed a single symmetric elution peak corresponding to the expected theoretical mass of 159 kDa due to its native trimeric arrangement. This value was also confirmed by laser scattering analysis which results suggested a globular structure with a diameter of 9–10 nm (Fig. 3C), in full accordance with the previously predicted theoretical size of the trimeric format in solution^8^.

### Functional *in vitro* characterization

**Figure 4:**
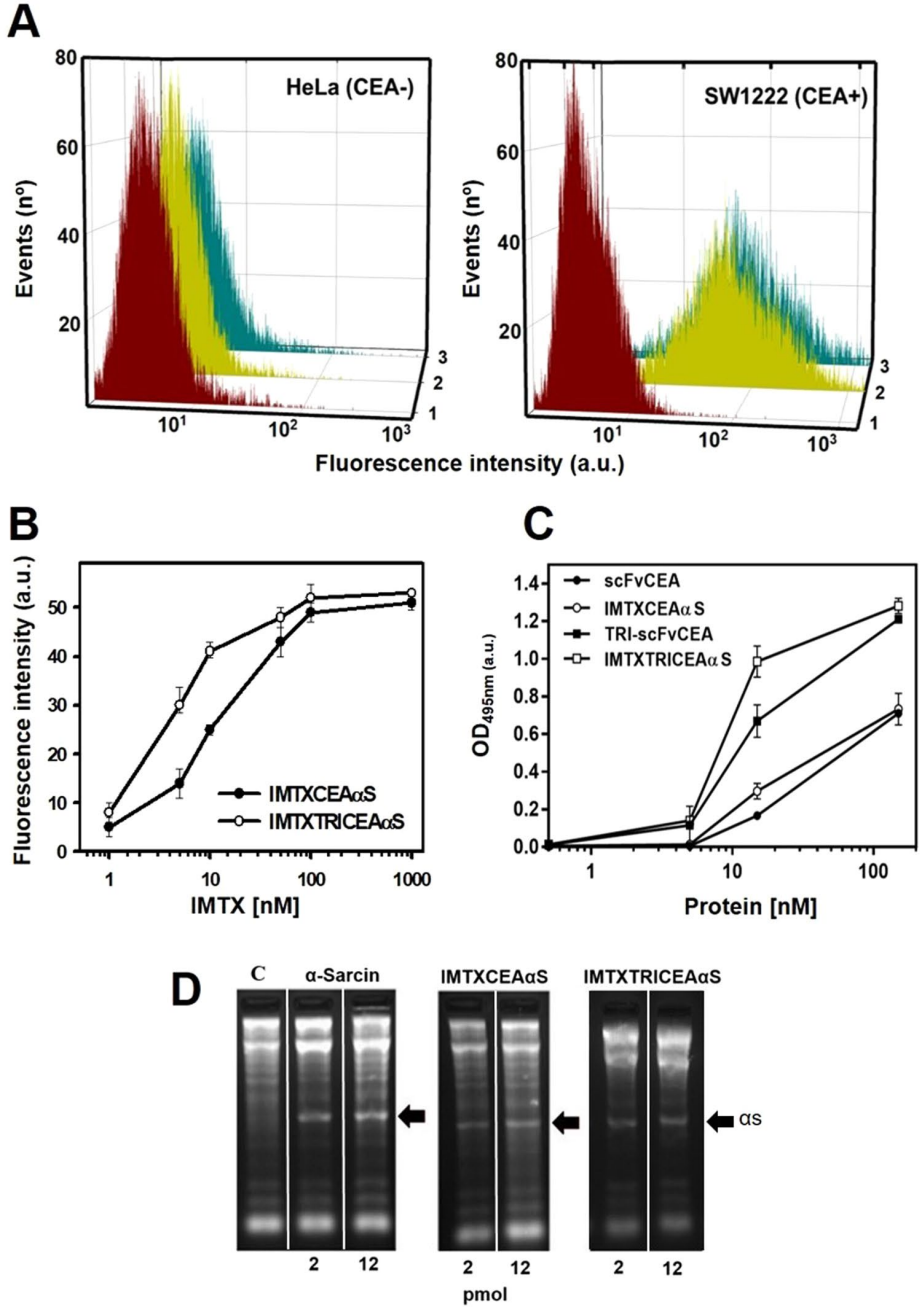
*In vitro* functional characterization. (**A**) Binding assay of the targeting domain by flow cytometry analysis with Hela (CEA-negative) and SW1222 (CEA-positive) cells. Curves correspond to cells incubated with (1) secondary antibody anti-His-Alexa-488; (2) IMTXCEAαS and (3) IMTXTRICEAαS. Fluorescence intensity was represented in arbitrary but proportional units. (**B**) Binding titration by flow cytometry using SW1222 cells incubated with different concentrations of IMTXCEAαS and IMTXTRICEAαS. (**C**) ELISA assay against immobilized CEA (0.25 µg/well). Titration was performed with serial dilutions of both immunotoxins including parental monomeric and trimeric versions of scFvMFE23. (**D**) Ribonucleolytic activity of the toxic domain: Rabbit reticulocytes assays were made in order to test the ribonucleolytic activity of α-sarcin within both constructs. The gels show the release of α-fragment, highlighted by a black arrow, produced by the specific SRL cleavage. In the example shown, 2 and 12 pmol were assayed for both immunotoxins and fungal wild-type α-sarcin. C, negative control where the protein sample was replaced by buffer. Images correspond to full-length gels and blots acquired and analyzed using the Gel Doc XR Imaging System and Quantity One 1-D analysis software (BioRad). Gels presented in the figure were cropped from from different gels or from different parts of the same gels. Original full-length gels are presented in Supplementary Fig. S2.

The first step in the functional characterization of both immunotoxins was to evaluate the ability of their targeting domain (MFE23 scFv) to bind CEA, either expressed on the cells surface or as a protein immobilized on plastic. According to flow cytometry results, both immunotoxin constructs were able to recognize CEA-positive SW1222 cells but not the CEA-negative HeLa cells (Fig. 4A). The trimeric immunotoxin recognize and bind CEA-positive SW1222 cells more efficiently than the monomeric design (Fig. 4B). In accordance with the flow cytometry analysis, ELISA binding titration curves revealed how the trimeric immunotoxin showed higher binding to immobilized CEA than the monomeric version (Fig. 4C).

Next, we analyzed the highly specific ribonucleolytic activity of the α-sarcin moiety. IMTXCEAαS and IMTXTRICEAαS were able to release the characteristic α-fragment, produced by the specific cleavage of a single phosphodiester bond at the rRNA sarcin-ricin loop (SRL), as efficiently as α-sarcin alone (Fig. 4D). Thereby, these results proved that the ribonucleolytic α-sarcin activity was preserved in both immunotoxins.

**Figure 5:**
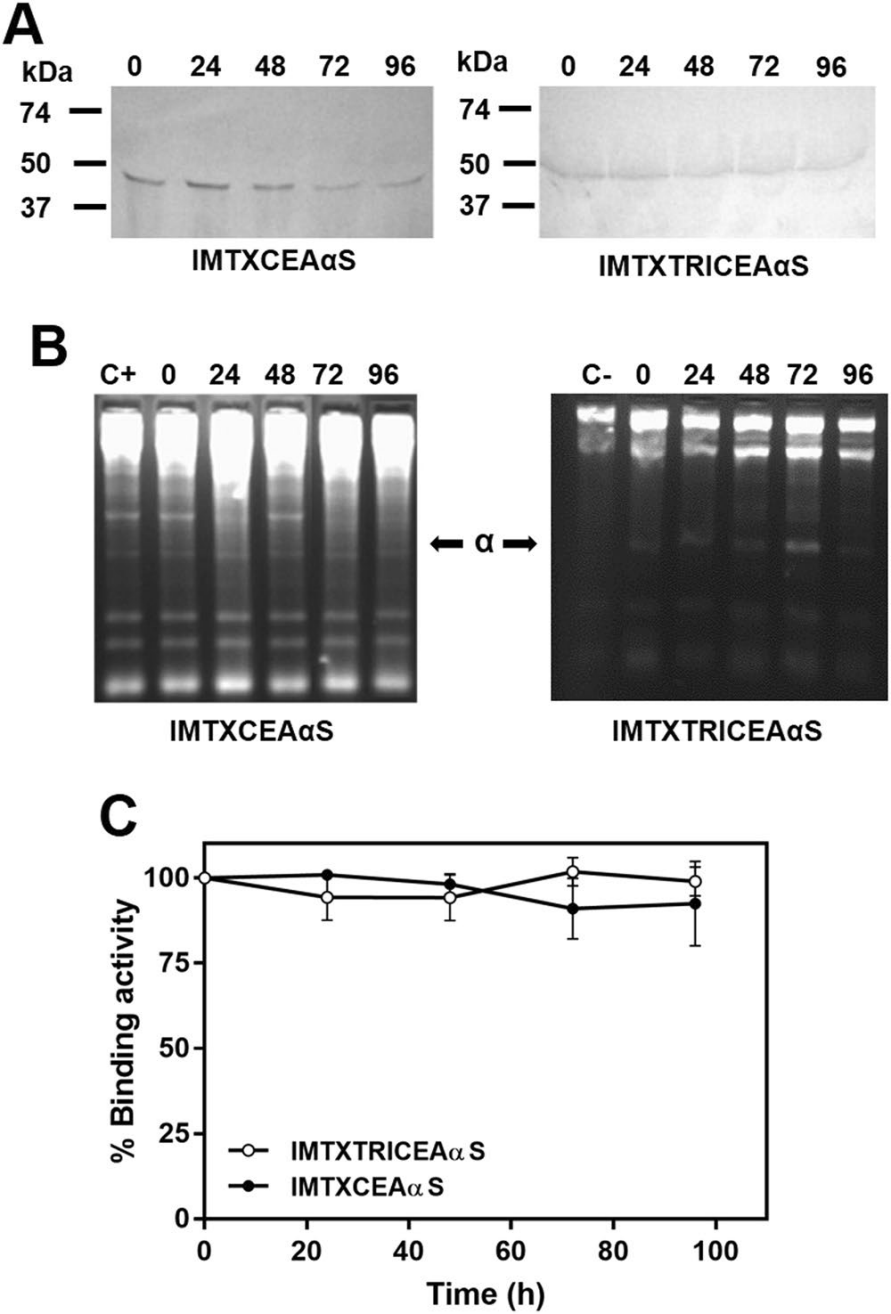
Immunotoxin stability at physiological conditions. Different aliquots of IMTXCEAαS and IMTXTRICEAαS were incubated with FBS at 37 °C for up to 96 hours. Aliquots were taken every 24 h and analyzed. (**A**) Western blot analysis using an anti-α-sarcin antisera. Cropped-blots are displayed. Full-length blots are presented in Supplementary Fig. S5A. Blot images were acquired and analyzed using the ChemiDoc-It (UVP) and VisionWorks LS (**B**) Ribonucleolytic activity assays. IMTXCEAαS gel displayed was cropped and reorganized from the original gel. Gel image was acquired and analyzed using the Gel Doc XR Imaging System and Quantity One 1-D analysis software (BioRad). The original full-length gel is presented in Supplementary Fig. S3B. (**C**) Flow cytometry study of immunotoxins binding to SW1222 cells.

In addition, both constructs exhibited high structural stability and maintained their functional integrity in conditions mimicking a physiological context. After incubation of the purified proteins in FBS for 96 hours at 37 °C, western blot analysis showed that both immunotoxins kept their full molecular integrity (Fig. 5A) for at least 48 hours. Furthermore, their ribonucleolytic activity (Fig. 5B) and CEA binding ability, as assessed by ELISA (Fig. 5C) were also preserved during that period of time when compared to freshly prepared control samples. It is important to note that even after 96 hours of incubation the binding activity of both constructs was still around 90% of the original value.

**Figure 6:**
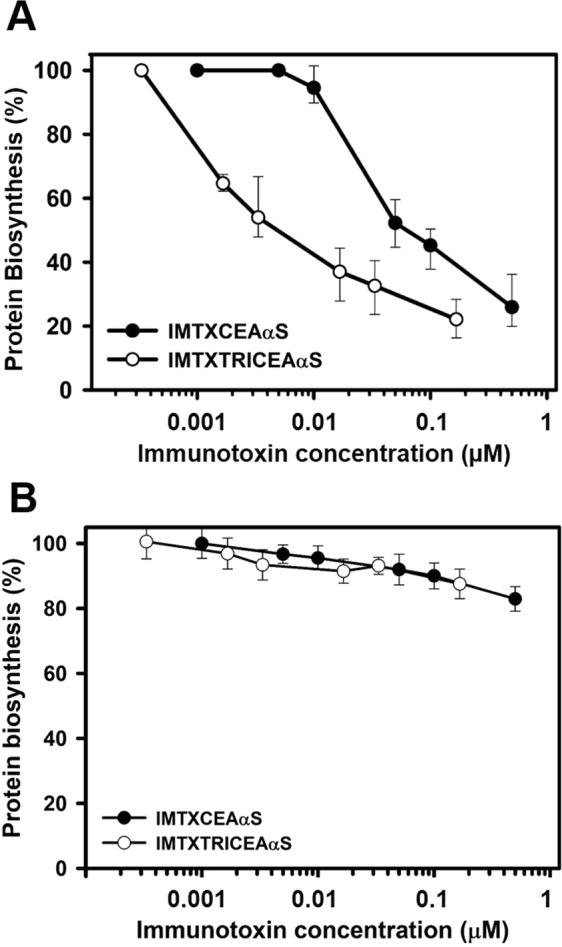
*In vitro* cytotoxicity characterization. Protein biosynthesis inhibition assay: CEA-negative cells HeLa (**A**) and CEA-positive cells SW1222 (**B**) were treated for 72 hours with both immunotoxin versions before addition of L-[4,5-^3^H]-Leucine. Protein concentrations are expressed in molar units, IMTXCEAαS (black circles) and IMTXTRICEAαS (white circles). Measurements were done and plotted (mean ± SD) referred to untreated controls. In all cases triplicates samples were used.

Once checked that both targeting domains and toxic payload not only kept their functional activities within the purified immunotoxins but also were stable for reasonably long periods of time in physiologically-relevant conditions, inhibition of protein synthesis was studied. After 72 hours of incubating CEA-positive SW1222 and CEA-negative HeLa cells with the immunotoxins, ^3^H-Leu incorporation was calculated as a measurement of protein biosynthesis (Fig. 6). As expected inhibition of protein synthesis was only observed within antigen-positive cells. These experiments allowed the calculation of an IC_50_ value for both immunotoxins as the molar concentration of protein needed to produce a 50% of protein synthesis inhibition. As shown in Fig. 6, IMTXTRICEAαS was significantly more effective in inhibiting protein synthesis in CEA-positive SW1222 (IC_50_ = 6 nM) than monomeric IMTXCEAαS (IC_50_ = 60 nM).

### *In vivo* antitumoral effect

**Figure 7:**
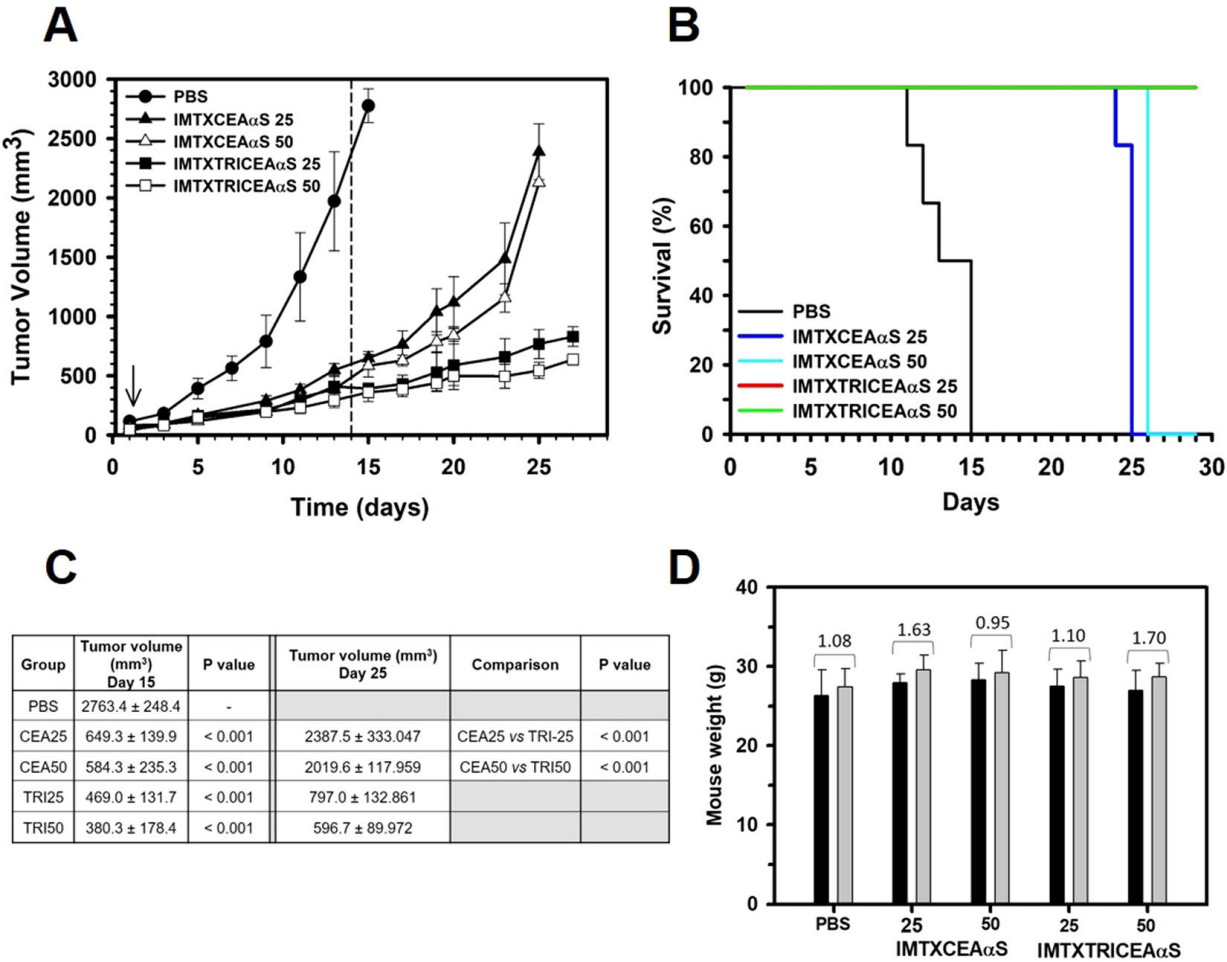
*In vivo* antitumoral activity. (**A**) Time course of tumor volume progression of SW1222-derived xenografts. Mice were non-treated (PBS) or treated with two different doses (25 or 50 μg) of IMTXCEAαS or IMTXTRICEAαS (labeled in the graph as IMTX 25 or 50, respectively) per injection. The arrow indicates the beginning of treatment. Doses were given every 48 hours. The dashed line indicates the end of treatment. (**B**) Statistical analysis of IMTXCEAαS and IMTXTRICEAαS-treated tumors *vs* vehicle-treated tumors at the end of the treatment (day 14) and IMTXCEAαS *vs* IMTXTRICEAαS-treated groups (day 25). (**C**) Kaplan-Meier survival curves: The Kaplan-Meier plots show the time to the experimental endpoint (once tumor volume reach 2000 mm^3^) of the *in vivo* assay. Last data used correspond to day 28 since the beginning of treatment. (**D**) Mice weight evolution: Weight measurement before and after treatment. Black bars (before treatment), grey bars (end of the treatment, day 14). Increase in weight (g) is indicated for each experimental group. No significant statistical differences were observed between the different experimental groups.

Finally, the antitumor activity of two different doses (25 or 50 µg) of IMTXCEAαS and IMTXTRICEAαS was assayed in *nude* mice bearing CEA-positive human colorectal cancer xenografts (Fig. 7A). Both immunotoxins strongly inhibited tumor growth in a dose-dependent manner as compared to the control group by the end of treatment (day 14). Remarkably, we observed in the IMTXCEAαS groups a rapid tumor growth rebound after the last injection, whereas mice receiving IMTXTRICEAαS showed long-lasting antitumor effect. In fact, at day 25 mean tumor volumes in the IMTXCEAαS groups were around three times larger than in the IMTXTRICEAαS ones (Fig. 7A,C). Consequently, mice treated with IMTXTRICEAαS showed a higher survival rate (i.e time to reach 2000 mm^3^; P = 0.0001), as shown in the Kaplan-Meier curves (Fig. 7B). Importantly, no signs of toxicity were observed at the doses used, considering body weight and external appearance of the mice (Fig. 7D).

## Discussion

Chimeric proteins combining antibody specificity for a user-defined TAA expressed on the cell surface and toxin-derived cytotoxicity (immunotoxins) are important tools for novel cancer therapy strategies. However, next-generation designs are necessary in order to overcome the limitations that currently restrict their therapeutic use, because of suboptimal tumor targeting, tumor penetration and biodistribution, or systemic side effects of non-targeted toxins^11,30^. Recently, the use of α-sarcin as an suitable toxic moiety in recombinant immunotoxins against colorectal cancer has been reported. This immunotoxin showed potent and specific antitumor effectiveness, due to a decrease in cancer cell proliferation and tumor angiogenesis and increased in apoptotic activity, while avoiding unspecific and cytotoxicity against normal cells^25,46^. At the same time, toxin accumulation within targeted cells was considerably increased by the high-affinity binding of the scFv antibody domain^16,25^.

Interestingly, novel multivalent antibody formats appear to increase immunotoxin tumor targeting capacity compared to the monovalent version. Indeed, trimeric immunotoxins are multivalent constructs showing increased functional affinity (avidity). Therefore, the use of these improved antibody formats for the design of next-generation immunotoxins seems to be a promising alternative. Hypothetically, this approach would further increase the toxic payload within the targeted cells and, consequently, it would reduce the amount of immunotoxin necessary to obtain identical antitumor effect along to the risk of undesired non-specific side effects.

As a proof of concept, we have generated, purified and characterized two different α-sarcin-based constructs. The main goal driving this approach was to assess the impact of the trimerbody format in an immunotoxin context, in terms of antigen binding and cytotoxicity. Both immunotoxins, were correctly produced and purified to homogeneity from *P*. *pastoris* extracellular media, after methanol induction. Both proteins showed the expected size, according to their monomeric or trimeric format, and CD spectra consistent with their globular β-sheet content. They were also able to specifically bind to CEA on the cell surface and to internalize into the target cells. Furthermore, they kept α-sarcin highly specific ribonucleolytic activity, needed for ribosome inactivation, and were stable and functional in physiological-like conditions. Therefore, it seemed safe to conclude that the immunotoxins, both the monomeric and trimeric versions, were properly folded, according to their structural and functional properties.

Their specific cytotoxic activity was evaluated *in vitro* against SW1222 cells, used as a model of CEA-positive colorectal cancer cell line, and against HeLa cells as off-target cell line. Although both immunotoxins inhibited protein biosynthesis specifically in CEA-positive cells, IMTXTRICEAαS promoted more efficient protein synthesis inhibition than the monomeric version. Thereby, the application of the trimerbody technology platform to α-sarcin immunotoxins improved its cytotoxic effect, at least *in vitro*. The observed reduction of the IC_50_ value for the trimeric immunotoxin would be attributed to its multivalence, increased avidity and higher total amount of toxin load inside the target cells.

The effect observed with both anti-CEA immunotoxins *in vitro* was also confirmed *in vivo* using *nude* mice bearing subcutaneous SW1222 xenografts. Both CEA-targeted immunotoxins caused a significant inhibition of tumor growth. In accordance with the *in vitro* protein biosynthesis inhibition assays, IMTXTRICEAαS also promoted higher inhibition of tumor growth. Furthermore, this effect was maintained for longer time, with a higher survival rate, once the treatment had finished. Mice treated with IMTXTRICEAαS showed a very slow tumor growth rate till at least day 30, when mice were sacrificed. The improved effect of IMTXTRICEAαS *in vivo* could also be attributed to a more favorable pharmacokinetics, in terms of half-life and tumor retention. In fact, it has just been reported a faster clearance of the monomeric MFE23 scFv compared to the corresponding trimerbody, which showed higher tumor accumulation in CEA-positive tumor-bearing mice^56^. Thus and as might be expected from its improved *in vivo* tumor targeting properties, the trimerbody-based immunotoxin showed to be much better therapeutic drug in terms of its antitumor effectiveness. Taking into account that multi-targeted therapies are currently considered as cutting-edge anti-tumoral strategies^58^, we could design trimeric immunotoxins with binding domains directed against different TAAs, increasing tumor specificity and avoiding tumor escape due to antigen-loss, to enhance the effectiveness of these molecules^59^.

Different therapeutic strategies and clinical trials have been developed using MFE-23, including the design of diabodies^60^, immunocytokines, BITEs (bispecific T cell engagers)^61,62^, antibody-directed prodrug therapy strategies^63^ and an anti-CEA chimeric antigen receptor (CAR). In this sense, T cells transduced to express this CAR, efficiently killed CEA+ cells *in vitro*, and inhibited the growth of CRC tumors *in vivo*, but unfortunately the trial was prematurely closed due to lack of prolonged CAR T cell persistence and acute respiratory toxicity^64^. Given that no MFE23-based therapeutic strategy is being pursued currently for the treatment of CEA+ solid tumors, we considered the existence of a window of opportunity for the design and characterization of MFE23-based immunotoxins.

In summary, the results herein presented not only confirm the potential of CEA-targeted ribotoxin-based immunotoxins, but also represent a further step in the development of next-generation biopharmaceuticals. To our knowledge, we have generated the first immunotoxin with trimeric format, and demonstrated its enhanced cytotoxic activity *in vitro* and therapeutic effect *in vivo* compared to the classical monomeric version. The modular design of the construct makes easy the substitution of MFE23 by other scFv directed against different TAA, so we can envision an array of ribotoxin-based immunotoxins for the treatment of a variety of cancers.

## Methods

### Plasmid construction

Plasmids encoding α-sarcin^25^, scFvMFE23 and the TIE^XVIII^ domain^8,27^, were previously obtained. The relevant cDNA sequences were amplified by PCR, including the restriction sites needed for cloning. The IMTXCEAαS or IMTXTRICEAαS cDNAs were cloned in pPICZαA (Invitrogen) for their extracellular production in the methylotrophic yeast *P*. *pastoris BG11*. The linker between the two domains of the monomeric immunotoxin was the tripeptide Gly-Gly-Arg. Regarding the trimeric immunotoxin, the sequence L18-TIE^XVIII^-L18 was inserted between the targeting and toxic domains. Finally, a six His-tag was added at the C-terminus of both immunotoxins for protein detection and purification (Fig. 1). The resulting plasmids were propagated in *Escherichia coli DH5αF’* and sequenced at the DNA sequence Genomics Unit of the Universidad Complutense.

### Protein production

Electrocompetent *P*. *pastoris BG11* cells were electroporated with 10 µg of linearized plasmid after digestion with *Pme* I, as previously described^25,38,45^, using a Bio-Rad Gene pulser apparatus. The yeast containing the desired constructions were selected after plating the electroporation mixture in YPDS plates with increasing amounts of zeocin (100–750 µg/ml). Multiple independent clones were tested in order to find the most productive colonies. For these screenings, cells were grown for 24 hours at 30 °C in BMGY in 24-well plates, being harvested afterwards by 10 min and 1600 g centrifugation. They were then suspended in BMMY for protein production induction at 25 °C and 200 rpm shaking. Protein production was periodically monitored up to 96 hours, adding methanol every 24 hours to a final concentration of 0.5% (v/v) to maintain a steady induction. Protein secretion to the extracellular media over time was analyzed by 0.1% (w/v) sodium dodecyl sulfate (SDS)-15% (w/v) polyacrylamide gel electrophoresis (PAGE) and western blot. A rabbit anti-α-sarcin serum was used for specific detection of the ribotoxin moiety. Given the location of the poly-His tag at the C-terminal end, the integrity of the purified protein was also checked by Western blot using a commercially available anti-histidine tag antibody. Before performing large-scale production, the protein synthesis levels of selected colonies were checked. Those ones with the highest production level were selected for each construction and stored at −80 °C in glycerol media for later use. For large-scale production, growing was performed for 24 hours at 30 °C and 200 rpm using five two-litter baffled Erlenmeyer flasks, each containing 400 ml of BMGY. Induction was carried out in the same Erlenmeyer flasks containing 200 ml of BMMY. Temperature was reduced to 25 °C, (maintaining the same shaking speed). After 24 hours of induction, the media was completely renewed by centrifugation and the cultured induced in the same conditions for another 24 hours. The extracellular medium was collected by centrifugation and dialyzed against 50 mM sodium phosphate buffer, containing 0.1 M NaCl, pH 7.5.

### Protein purification

Both immunotoxins were purified from the dialyzed extracellular medium using a Ni^2+^-NTA agarose column (HisTrap^TM^ FF Columns, GE Healthcare). The medium was applied on the column at 1 ml/min using a peristaltic pump. The column was first washed with 50 mM sodium phosphate buffer, 0.1 M NaCl, pH 7.5, followed with the same buffer containing 20 mM imidazole. Finally, the immunotoxins were eluted rising imidazole to 250 mM. Fractions containing the purified protein were pooled and exhaustively dialyzed against the 50 mM sodium phosphate buffer, 0.1 M NaCl, pH 7.5.

### Biophysical characterization

Immunotoxins structural characterization was performed as previously described^19,25,38,45^. Absorbance measurements were carried out on an Uvikon 930 spectrophotometer (Kontron Instruments). Far-UV circular dichroism (CD) spectra were obtained from immunotoxin samples at 0.15 mg/ml in 50 mM sodium phosphate buffer, 0.1 M NaCl, pH 7.5, using a Jasco 715 spectropolarimeter and a scanning speed of 50 nm/min. Cells of 0.1-cm optical path were employed. Four spectra were averaged to obtain the final data.

To check the globular size of both immunotoxins in solution, FPLC was performed using a Superdex 200 column (GE Healthcare Life Sciences) in an AKTA purifier apparatus (GE Healthcare Life Sciences). With the same purpose, laser scattering measurements with the native trimeric immunotoxin in solution were also made at the Spectroscopy and Correlation Facility of the Universidad Complutense. Briefly, two different concentrations of IMTXTRICEAαS, 0.15 and 0.3 mg/ml, were filtered through 0.22 µm filters and analyzed at 25 °C for 60 seconds. The resulting size distribution curves were recorded for size determination, using the average value of ten spectra. Data were analysed using Zetasizer Ver. 7.02 software.

### Ribonucleolytic activity assays

The highly specific ribonucleolytic activity of α-sarcin was tested against ribosomes from a rabbit cell-free reticulocyte lysate as previously described^25,45^. The release and detection of a characteristic 400 nt rRNA fragment, known as α-fragment, shows unambiguously the α-sarcin activity against the SRL With this purpose, the lysate was first diluted 3-fold with 40 mM Tris–HCl, pH 7.5, containing 40 mM KCl and 10 mM EDTA. Then, 50 μl aliquots of this dilution (containing 5–6 pmol of ribosomes approximately) were incubated for 15 min at room temperature with different amounts of the tested proteins. The reaction was stopped by addition of 250 μl of 50 mM Tris–HCl, pH 7.4, containing 0.5% (w/v) SDS, followed by strong vortexing. Then RNA phenol/chloroform extraction was performed and the RNA was precipitated from the aqueous phase by adding isopropanol. Finally, the resulting pellet was washed with 70% (v/v) −20 °C ethanol, air dried and resuspended in 10 μl of DEPC H_2_O. α-Fragment release was visualized, after heating the samples at 90 °C for 5 min, by electrophoresis on denaturing 2% agarose gels and ethidium bromide staining.

### Cell Lines growth and culture

HeLa cells (human cervix adenocarcinoma; CCL-2), obtained from the American Type Culture Collection (Rockville, MD, USA), were used as tumoral CEA-negative cells, whereas colon carcinoma SW1222 cells, provided by Dr. Carl Batt under the partnership Cornell University-Ludwig Institute of Cancer Research, were used as model for the positive ones. HeLa cells were grown as described^47,65^, in Dulbecco’s modified Eagle’s medium; meanwhile SW1222 cells were maintained in RPMI-1640 medium^25^. Both media contained 300 mg/ml of L-glutamine, 50 U/ml of penicillin, and 50 mg/ml of streptomycin, and were supplemented with 10% fetal bovine serum. Incubation was performed at 37 °C in a humidified atmosphere (CO_2_:air, 1:19, v-v). Harvesting and propagation of cultures were routinely performed by trypsinization. The number of cells was determined using a hemocytometer.

### Flow cytometry

Trypsinized cells were distributed into aliquots of 3 × 10^5^ cells and washed three times with phosphate buffered saline (PBS). These aliquots were incubated with different concentrations of purified immunotoxins, using scFvMFE23 as a positive control, for 1 h at room temperature with gentle agitation. A second incubation was performed in the dark with anti-His-Alexa488 (Sigma) diluted 1/100. Between incubations and after the final one, the cells were sedimented by centrifugation (1200 g, 4 °C, 10 min) and washed with PBS for three times. Flow cytometry was performed on a FACScan (Becton Dickinson) and data were analyzed using the WinMDI software.

### ELISA

The ability of IMTXCEAαS, IMTXTRICEAαS and their scFv counterparts to bind human CEA was studied by ELISA as previously described^27^. Plates (Nunc A/S. Roskilde, Denmark) were coated with CEA (0.25 µg/well), washed and blocked with 5% BSA in PBS. Then, 100 µl with the corresponding concentration of the different constructions were added and incubated for 1 hour at room temperature. After three washes, the wells were incubated for one more hour at room temperature with an anti His-tag antibody (BioRad). Washes were repeated and HRP-conjugated goat anti-mouse antibody was added for another hour at room temperature. Then, the plate was washed and developed with the corresponding substrate. Antigen-binding titration was performed with serial dilutions of the purified immunotoxins. Three independent replicates were conducted to calculate the average values.

### Protein biosynthesis inhibition

As previously described^47–49^, protein biosynthesis inhibition is the assay routinely used to evaluate the toxic effect of ribotoxins. To evaluate the cytotoxic effect of both immunotoxins, cells were seeded into 96-well plates (1 × 10^4^ cells/well) in culture medium and maintained under standard culture conditions for 24 hours. Then 200 µl of free-FBS fresh medium with different immunotoxin concentrations were added. Following 72 hours of incubation at 37 °C, the medium was removed and replaced with fresh one supplemented with 1 mCi per well of L-[4,5–3 H]-Leucine (166 Ci/mmol; Amersham, UK). After 6 hours, medium was removed and cells were fixed with 5.0% (w/v) trichloroacetic acid. The resulting pellet was finally washed three times with cold ethanol and dissolved in 200 µl of 0.1 M NaOH containing 0.1% SDS. Its radioactivity was counted on a Beckman LS3801 liquid scintillation counter. The results were expressed as percentage of the radioactivity incorporated to calculate IC_50_ values (protein concentration inhibiting 50% protein synthesis). Three independent replicates per two assays were conducted to calculate the average IC_50_ values.

### Serum stability assays

For testing the structural and functional stability of the new recombinant designs, both immunotoxins were incubated in 60% (v/v) FBS during 96 hours at 37 °C in sterile conditions at a final concentration of 100 nM. Every 24 hours an aliquot was removed and quickly frozen at −80 °C until the entire study was completed. As a control, serum-exposed immunotoxins were frozen immediately to represent a zero time point. Identical aliquots of the different time-points samples were tested for their capability to bind CEA by ELISA and their ribonucleolytic activity. In addition, the immunotoxin molecular integrity was tested by anti-α-sarcin western blot.

### Xenograft tumor growth inhibition on nude mice

All animal procedures were performed with the approval of the Universidad Complutense Animal Experimentation Committee, according to the European official regulations. Balb/c *nude* male mice (7 weeks old) were purchased from Harlan Laboratories S.A. (Barcelona, Spain) to evaluate the *in vivo* effect of IMTXCEAαS and IMTXTRICEAαS against human colorectal cancer xenografts. Studies were performed in the Animal Facility of the Centro de Investigaciones Biológicas-Consejo Superior Investigaciones Científicas (CIB-CSIC) in Madrid.

Mice were allocated into five experimental groups (*n* = 6): PBS (phosphate buffered saline), IMTXCEAαS or IMTXTRICEAαS treated with 25 or 50 µg of immunotoxin per injection (IMTX 25 or 50, respectively). Prior to the experiments, animals were given a 7-day adaptation period with free access to food and water. Each mouse received a subcutaneous injection into the right flank of 2 × 10^6^ SW1222 cells, resuspended in 200 µl of 1:1 PBS-Matrigel (BD Biosciences) mixture. Once the tumor volume reached 50–100 mm^3^, mice were injected intraperitoneally either with PBS or the different immunotoxins. Seven doses, every 48 hours, of PBS or the two different amounts of immunotoxin (25 or 50 µg) were given.

Tumors were routinely measured with an external caliper, and volume was calculated as (width/2)^2^ × (length/2). Mice were also weighted throughout the experiment. At the end of the treatment (15^th^ day), or before in case of potential suffering, animals from control group were sacrificed and tumors were collected for further analysis. After the last treatment dose (15^th^ day) in immunotoxins-treated groups, the evolution of tumor growth was followed in four mice randomly selected from each group, while the other two mice were sacrificed for comparison with the control group if necessary.

### Statistical analysis

ANOVA with a post hoc analysis by the Student-Newman-Keuls’ test was used to compare variations in the mean tumor sizes at different treatment time points in each experimental group. Differences between experimental groups were considered statistically significant at P < 0.05. All values were expressed as arithmetic means ± sem (standard error of the media).

### Equipment and settings

Gels images from Figs 2A, 4D and 5B were acquired and analyzed using the Gel Doc XR Imaging System and Quantity One 1-D analysis software (BioRad).

Blots images from Figs 2B and 5A were acquired and analyzed using ChemiDoc-It (UVP) and VisionWorks LS analysis software.

If processing in brightness and contrast of gel and blot images has been made it was applied to the entire image including controls. No high-contrast gels or blots has been displayed. When necessary, cropped gels and juxtaposing images were displayed to improve the clarity and conciseness of the presentation, being explicited in the figure.

SigmaPlot - Scientific Data Analysis and Graphing Software (Systat Software Inc.) was used for statystical analysis an graphing of experimental data in Figs 3, 4A–C, 5C, 6 and 7.

## Supplementary information

Supplementary Figures and captions

## Data Availability

All data generated or analysed during this study are included in this published article.
