# Supplementary material for: A novel Carcinoembryonic Antigen (CEA)-Targeted Trimeric Immunotoxin shows significantly enhanced Antitumor Activity in Human Colorectal Cancer Xenografts
Source: Sci Rep. 2019 Aug 12;9:11680. doi: 10.1038/s41598-019-48285-z (PMC6690998; doi:10.1038/s41598-019-48285-z)
Supplement: Supplementary file 1 — Supplementary Figures and captions [file 41598_2019_48285_MOESM1_ESM.pdf]

A novel Carcinoembryonic Antigen (CEA)-Targeted Trimeric Immunotoxin shows significantly enhanced Antitumor Activity in Human Colorectal Cancer Xenografts

Lázaro-Gorines, R.

Ruiz-de-la-Herrán, J.

Navarro, R.

Sanz, L.

Álvarez-Vallina, L.

Martínez-del-Pozo, A.

Gavilanes, JG.Lacadena, J.

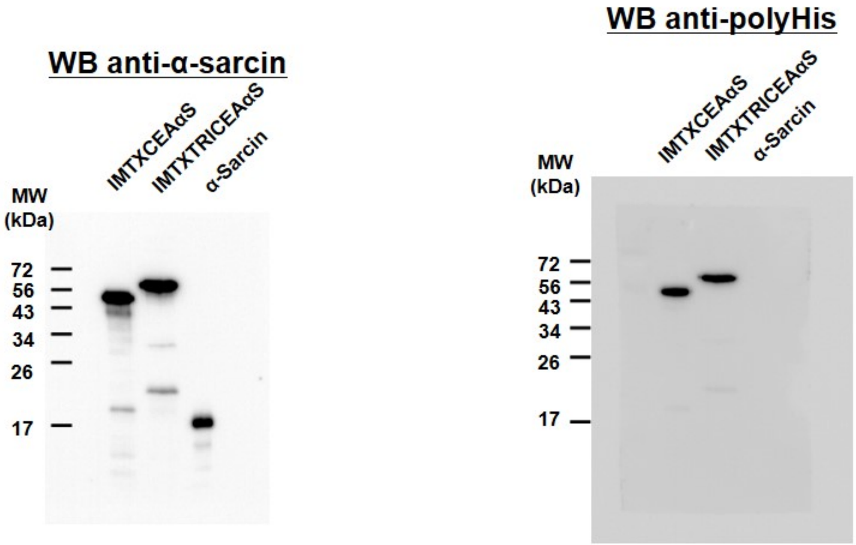
WB anti-α-sarcin
IMTXCEAαS
IMTXTRICEAαS
α-Sarcin
MW
(kDa)
72
56
43
34
26
17
WB anti-polyHis
IMTXCEAαS
IMTXTRICEAαS
α-Sarcin
MW
(kDa)
72
56
43
34
26
17

Figure S1

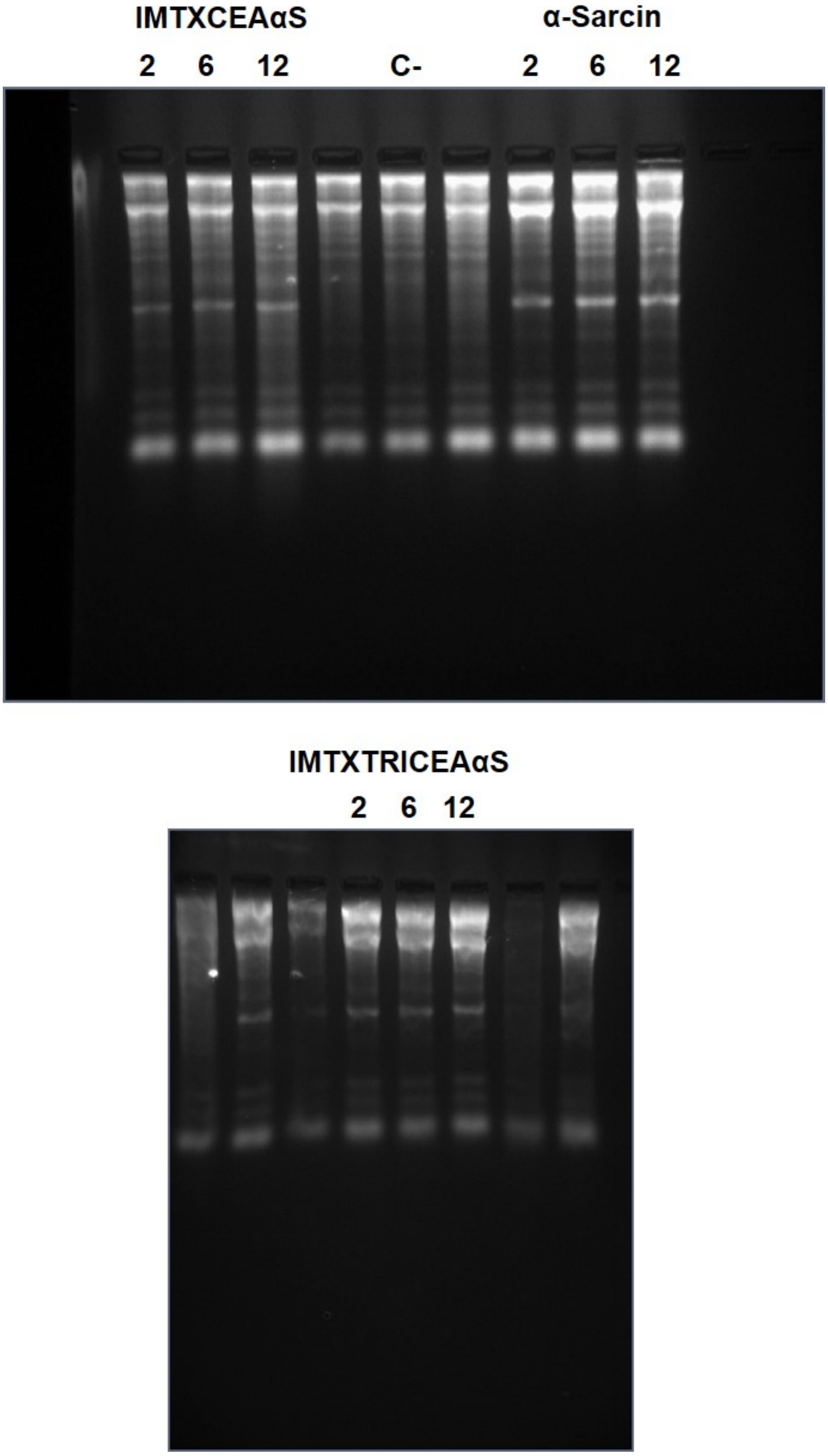

Figure S2

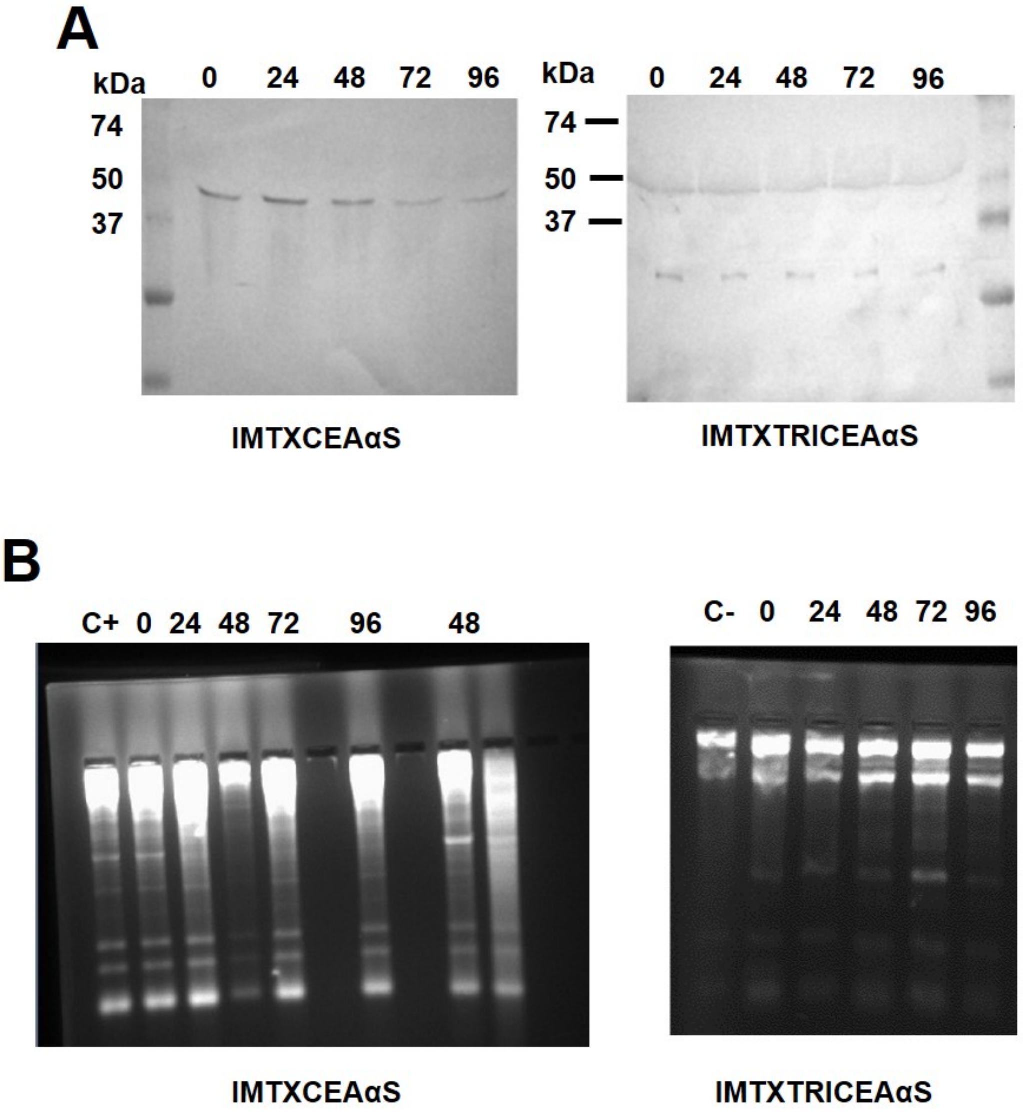
A
kDa
0
24
48
72
96
74
50
37
IMTXCEAαS
kDa
0
24
48
72
96
74
50
37
IMTXTRICEAαS
B
C+
0
24
48
72
96
48
IMTXCEAαS
C-
0
24
48
72
96
IMTXTRICEAαS

Figure S3

Supplementary Figures Legends

Figure-S1. SDS-PAGE and Western Blot analysis of both purified immunotoxins. A) Western blot analysis using rabbit anti-α-sarcin serum (left) or commercial anti-histidine tag antibody (right). α-Sarcin designates 0.1 µg of the fungal natural protein used as a control. MW corresponds to prestained Bio-Rad Precision Plus protein molecular weight standards. Images correspond to full-length gels and blots acquired and analyzed using the Gel Doc XR Imaging System and Quantity One 1-D analysis software (BioRad) or ChemiDoc-It (UVP) and VisionWorks LS, respectively. In this figure different exposure of the blots presented in Figure 2 are shown.

Figure-S2. ***In vitro* functional characterization.** Ribonucleolytic activity of the toxic domain: Rabbit reticulocytes assays were made in order to test the ribonucleolytic activity of α-sarcin within both constructs. Original full-length gels from Figure 4D are presented. The gels show the release of α-fragment, highlighted by a black arrow, produced by the specific SRL cleavage. In this origjnal full-length gels, 2, 6 and 12 pmol were assayed for both immunotoxins and fungal wild-type α-sarcin. C, negative control where the protein sample was replaced by buffer. Gel images were acquired and analyzed using the Gel Doc XR Imaging System and Quantity One 1-D analysis software (BioRad).

Figure-S3. **Immunotoxin stability at physiological conditions**. Different aliquots of IMTXCEAαS and IMTXTRICEAαS were incubated with FBS at 37ºC for up to 96 hours. Aliquots were taken every 24 h and analyzed. A) Western blot analysis using an anti-α-sarcin antisera. Full-length blots from Figure 5A are displayed. Blot images were acquired and analyzed using the ChemiDoc-It (UVP) and VisionWorks LS B) Ribonucleolytic activity assays. IMTXCEAαS and IMTXTRICEAαS original full-length gels from Figure 5B are displayed. Gel image was acquired and analyzed using the Gel Doc XR Imaging System and Quantity One 1-D analysis software (BioRad).
